# Exploring the application of poly(1,2-ethanediol citrate)/polylactide nonwovens in cell culturing

**DOI:** 10.3389/fbioe.2024.1332290

**Published:** 2024-03-15

**Authors:** Aleksandra Bandzerewicz, Joanna Howis, Kamil Wierzchowski, Miroslav Slouf, Jiri Hodan, Piotr Denis, Tomasz Gołofit, Maciej Pilarek, Agnieszka Gadomska-Gajadhur

**Affiliations:** ^1^ Faculty of Chemistry, Warsaw University of Technology, Warsaw, Poland; ^2^ Faculty of Chemical and Process Engineering, Warsaw University of Technology, Warsaw, Poland; ^3^ Institute of Macromolecular Chemistry, Czech Academy of Sciences, Prague, Czechia; ^4^ Laboratory of Polymers and Biomaterials, Institute of Fundamental Technological Research, Polish Academy of Sciences, Warsaw, Poland

**Keywords:** citric acid, citrate-based polyesters, biomaterials, nonwovens, electrospinning

## Abstract

Biomaterials containing citric acid as a building unit show potential for use as blood vessel and skin tissue substitutes. The success in commercializing implants containing a polymer matrix of poly(1,8-octanediol citrate) provides a rationale for exploring polycitrates based on other diols. Changing the aliphatic chain length of the diol allows functional design strategies to control the implant’s mechanical properties, degradation profile and surface energy. In the present work, poly(1,2-ethanediol citrate) was synthesized and used as an additive to polylactide in the electrospinning process. It was established that the content of polycitrate greatly influences the nonwovens’ properties: an equal mass ratio of polymers resulted in the best morphology. The obtained nonwovens were characterized by surface hydrophilicity, tensile strength, and thermal properties. L929 cell cultures were carried out on their surface. The materials were found to be non-cytotoxic and the degree of porosity was suitable for cell colonization. On the basis of the most important parameters for assessing the condition of cultured cells (cell density and viability, cell metabolic activity and lactate dehydrogenase activity), the potential of PLLA + PECit nonwovens for application in tissue engineering was established.

## 1 Introduction

Significant advances in the healthcare field, associated with faster diagnosis and improved surgical procedures, have increased the life expectancy of the population. As a result, susceptibility to disease and the risk of organ failure has risen. In response to the problem of donor shortage and the ineffectiveness of conventional pharmacological treatment, tissue engineering was developed. It combines medicine, biological sciences and material engineering to create functional substitutes for damaged tissues (Griffith and Gail, 2002; Ikada, 2006; Sharma et al., 2019).

The basic premise of tissue engineering is the regeneration of the patient’s tissues and organs using a scaffold that supports proliferating cells and shapes them as desired. The therapy is intended to use cells taken from the patient and replicated in an *in vitro* environment. In some cases, treatment can be limited to using an isolated scaffold, e.g., by applying a collagen sheet. Fibroblasts from the healthy tissue surrounding the damaged area will migrate into the pores of the sheet and secrete skin-building proteins and polysaccharides. At the same time, the scaffold will degrade into products absorbed and metabolized by the body (Ikada, 2006; Chaudhari et al., 2016; Caddeo, Boffito, and Sartori, 2017; Meyer, 2019; Sharma et al., 2019; Qi et al., 2022).

A potential benefit of using biodegradable polyesters in tissue engineering is the controllability of the crosslinking degree of the polymer chains. The aim is to obtain a mechanomimetic environment reflecting the intended target tissues. Furthermore, one can modulate the biomaterial’s chemical functionality and degradation rate by the degree of crosslinking. In a dynamic environment, elastomers show the post-implantation ability to return to their original shape after deformation without causing significant irritation to the surrounding tissue. The mechanical interaction between the implanted biomaterial and the native tissue must not cause inflammation since it limits the recovery of damaged tissue and leads to scar formation (He and Matsuda, 2002; Xue and Greisler, 2003; Webb, Yang, and Ameer, 2008). Numerous studies on the functionalization of citric acid polyesters have identified two basic crosslinking pathways: thermal (via esterification and click reactions) and by using crosslinking agents (Wang et al., 2022).

Fruit acids have broad applicability in various industrial sectors, particularly food, pharmaceuticals and cosmetics. The most commonly used is citric acid, for which the global market size reached 2.8 million tons as of 2022 (ltd n.d.). The multi-functionality of citric acid provides four key benefits related to the functional design strategy of biomaterials (Tran, Yang, and Ameer, 2015). (1) By thermal polycondensation with polyhydroxy alcohols, citric acid forms ester bonds that are susceptible to hydrolysis and facilitate biomaterial degradation. (2) The unreacted carboxyl and hydroxyl groups of the prepolymer provide the inherent bioactivity of the material and the necessary functionality for thermal crosslinking in subsequent post-polycondensation. (3) The controlled synthesis of the prepolymer allows for the preservation of an adequate amount of free functional groups, which are essential for the functionalization of the biomaterial and to ensure its antioxidant, antimicrobial or fluorescent properties. (4) The possibility of forming intermolecular hydrogen bonds affects the material’s mechanical properties (Tang et al., 2015; H; Zhao and Ameer, 2009; Gyawali et al., 2010; Guo et al., 2017; 2016).

Currently, much focus is directed towards using polylactide (PLA) for medical applications (Lumiaho et al., 1999; Gerber and Gogolewski, 2002; Tyler et al., 2016; Singhvi, Zinjarde, and Gokhale, 2019; VeliogluBusra et al., 2019; Arif et al., 2022). The limitation may be its hydrophobicity and long degradation time (DeStefano, Khan, and Tabada, 2020). The challenge is to obtain materials that support implant vascularization and promote cell adhesion and proliferation, hence the growing interest in citric acid-based polyesters. Among the best studied and described in the literature is poly(1,8-octanediol citrate) (POC), first reported in 2004 (Yang, Webb, and Ameer, 2004). The use of POC as a biomaterial has already progressed beyond the laboratory stage. Acuitive Technologies launched a technology for orthopedic surgery applications based on poly(1,8-octanediol citrate) in September 2021. CITREGEN^®^ is a ceramic-polymer composite containing phosphate and calcium molecules, essential for bone regeneration, and a polymer matrix providing structural integrity with the tissue. The technology has found application in a whole range of orthopedic implants for the fixation of connective tissue and, more specifically, ligaments and tendons in surgeries of the shoulder, elbow, knee and wrist (Qiu et al., 2006).

Poly(1,2-ethanediol citrate), the subject of this work, has not been reported in the literature so far. Compared to POC, the resulting material is expected to have higher stiffness and hydrophilicity, and the degradation time will be shortened. This is due to the shorter diol chain. As a result, the cross-linking density of the polycitrate will be higher, in the sense that the acid-derived branching centers will occur at shorter distances from each other. This will translate into a more rigid structure. For the same reason, the hydrophilicity of the material should increase - the hydrophilic groups responsible for this (in this case, all oxygen-containing moieties), will occur at shorter intervals in the polymer structure, replacing the long hydrophobic carbon aliphatic chains of POC. Due to its properties, the possibility of using it in nonwoven form as a substitute for blood vessels or skin tissue is postulated.

## 2 Materials and methods

### 2.1 Synthesis procedure

1,2-ethanediol (Fisher Chemical, ≥99%), anhydrous citric acid (Acros Organics, ≥99.5%) and p-toluenesulfonic acid (PTSA; Sigma Aldrich, ≥98.5%) monohydrate were used without prior preparation.

The synthesis of poly(1,2-ethanediol citrate) was carried out in a Mettler Toledo MultiMax parallel reactors system, using Hastelloy reactors (50 mL working capacity) with a Teflon cover equipped with a mechanical stirrer, temperature sensor, and DeanStark apparatus. The reaction mixture was heated up to 140°C for 10 min, and the temperature was then held constant for 50 min.

This synthesis was previously reported and verified (Howis et al., 2023).

### 2.2 Electrospinning

Poly(1,2-ethanediol citrate) was mixed with poly-L-lactide (PLA, Purasorb PL49, Corbion) to prepare a solution of the polymers in 1,1,1,3,3,3-hexafluoro-2-propanol (HFIP, Iris Biotech) at a mass concentration of 5%. The PECit content of the polymer mixture was 25, 50% and 75%. The solution was stirred for 24 h at a magnetic stirrer speed of 150 rpm. A rotational collector with a low linear speed (1 m/s) was used for the electrospinning, with a needle-collector distance of 14 cm and a solution feed rate of 1.5 mL/h. The nonwovens were dried to remove residual HFIP.

### 2.3 SEM imagining

Samples (small pieces of thin nonwoven films) were fixed to an aluminium support with a double-adhesive carbon tape and coated with a thin platinum layer (4 nm of Pt; vacuum sputter coater SCD 050; Leica, Vienna, Austria) to minimize a possible e-beam damage. The top surface of the samples was observed in an SEM microscope MAIA 3 (TESCAN, Brno, Czechia). All micrographs were obtained with a secondary electron detector at an acceleration voltage of 3 kV.

### 2.4 Tensile strength testing

Tensile testing measurements were performed with a universal testing machine (Instron 6025/5800R; Instron, High Wycombe, UK). The measurements were made with a 10 N load cell and grips with pneumatic rubbered clamps. Materials were cut into 5 mm × 35 mm pieces. The gauge length was fixed to 20 mm. A strain rate of 5 mm/min was applied. Five samples of the same material were used, the results were averaged, and the standard deviation values were calculated.

### 2.5 Hydrophilicity

The hydrophilicity of the nonwoven surface was determined by the water contact angle values using the sitting drop method. Samples were placed on the movable table equipped with the Digital Camera Industrial Digital Camera UCMOS01300KPA with Fixed Microscope Adapter FMA037. Tiny drops of liquid were placed onto the samples. ToupView software was used to analyze the shape of the droplets. Five measurements were taken for each tested material, the results were averaged, and standard deviation values were calculated.

### 2.6 Differential scanning calorimetry

Measurements were carried out on a TA Instruments Q2000 flow calorimeter. A temperature programme was established in heating-cooling-heating mode within a temperature range of −50°C to 250°C with a temperature step of 10°C/min under the nitrogen flow.

The enthalpy of fusion was determined, and the degree of crystallinity (*X*_c_) was calculated according to the formula:

$$X_{c}=\frac{{∆H}_{f}}{{∆H}_{f}^{0}}\times 100\%$$

where:

Δ*H*_f_–enthalpy of fusion of the sample, J/g,
Δ*H*_f_^o^–enthalpy of fusion of 100% crystalline PLLA, 93,6 J/g.

### 2.7 XTT cytotoxicity testing

L929 mouse fibroblasts (ATCC) were selected as model cells for culture. All cell procedures were performed in a laminar flow cabinet, sterilized with UV light prior to use. Studies were preceded by radiation sterilization of the materials. An electron accelerator ELEKTRONIKA 10/10 was used, and the electron energy was 9.1 MeV. A radiation dose of 15.0 kGy was applied.

Two Dulbecco’s Modified Eagle Medium (DMEM, Gibco) media varying in glucose concentration (1 g/L for cytotoxicity tests and 4.5 g/L for long-term cell culture) were independently used. Both media were supplemented with 10% v/v inactivated fetal bovine serum (Gibco) and 1% v/v Pen-Strep (Gibco).

Discs of 15 mm diameter were cut from the nonwovens and in 24-well plate, four discs a well. 1.3 mL of culture medium was poured into each well to prepare the extracts. The plate was incubated at 37°C for 24 h.

A cell suspension of 10^5^ cells/mL was placed in a 96-well plate, 100 μL per well, and incubated at 37°C for 24 h. The medium was removed, and the extracts were added, 100 μL per well. Some wells were flooded with fresh medium (negative control) or 0.1% Triton X-100 solution in DMEM (positive control). The plate was then incubated at 37°C for 24 h in an air atmosphere enriched with 5% CO_2_. Next, the culture medium was removed, and the cells were washed twice with 100 μL of DPBS. A 33% v/v of XTT reagent (CyQUANT XTT Cell Viability Assay, ThermoFisher Scientific) in DMEM was added, 150 μL per well. The plate was placed in an incubator (37°C) for 4 h. The absorbance was measured in the multiwell plate reader for 450 and 660 nm. Results were compared to the negative control, which was assumed to be 100% of the metabolic activity of cells.

### 2.8 L929 cell cultures

Discs of 1.5 cm diameter were cut from the nonwovens and placed in 24-well plates, one disc a well. 1.5 mL of inoculum (10^5^ cells/mL) was poured into each well. The plates were transferred to an incubator (37°C, 5% CO_2_). L929 cell cultures were carried out for 8 days without medium exchange. Zero-day analyses were performed after 4 h of incubation; other samples were harvested every 48 h. The following tests were carried out: cell density and viability assessment (trypan blue dye), metabolic activity (Presto Blue assay), and lactate dehydrogenase activity (LDH assay). Confocal microscopy (Carl Zeiss AG) was used to visualize the morphology of L929 cells.

#### 2.8.1 Cell density and viability

L929 cells were detached from the surface (by typical trypsinization) and the culture medium was aseptically pipetted-out from each well. The cells remaining on the nonwovens were rinsed twice with DPBS, no calcium, no magnesium buffer. 0.5 mL of 0.05% trypsin-EDTA was poured into each well. The plates were incubated at 37°C for 5 min 0.5 mL of fresh DMEM medium was added to inhibit trypsin and preserve suspended cells from trypsin-based digestion. Cell density and viability were determined by counting cells stained with 0.4% trypan blue aqueous solution (Thermo Fischer Scientific, US) using a Burker Turk hemocytometer (Brand, DE) and an Eclipse TS100 inverted microscope (Nikon, JP). Counting the total number of cells and the number of dead cells was repeated five times.

#### 2.8.2 Metabolic activity

Metabolic activity assessment was based on the PrestoBlue assay results (Thermo Fischer Scientific, US). 156 μL of PrestoBlue reagent was added to 1.5 mL of L929 cells culture or cell-free culture (reference). All samples were incubated at 37°C for 2 h. Then, absorbance was measured using a GENESYS 20 UV−VIS spectrophotometer (Thermo Fisher Scientific, US) at 570 and 600 nm. Metabolic activity was calculated according to the formula:

$$\text{metabolic activity}=37.04\times \left(A_{570}-A_{570REF}\right)-\left(A_{600}-A_{600REF}\right),\left[\mu \text{kat}/{\text{dm}}^{3}\right]$$

where:

*A*_570_—the absorbance of the test sample at 570 nm, *A*_570REF_–the absorbance of the reference sample at 570 nm, *A*_600_—the absorbance of the test sample at 600 nm, *A*_600REF_–the absorbance of the reference sample at 600 nm.

Results from three trials were averaged. The value of the standard deviation was calculated.

#### 2.8.3 LDH activity measurement

LDH activity has been determined according to the procedure of enzyme-based BioMaxima-LDH assay (BioMaxima, PL). Biomaxima-LDH reagent was prepared by mixing R1 and R2 reagents in a 3:1 volume ratio prior to use and then poured into the standard disposable spectrophotometric cuvettes, 1.0 mL per cuvette. 20 μL of culture medium samples filtered through syringe filters (ϕ = 0.2 μm) were added and mixed. Absorbance measurement was performed at 340 nm in 1-min intervals (from 0 to 3 min) using a GENESYS 20 UV−VIS spectrophotometer (Thermo Fisher Scientific, US). The values of LDH activity were calculated based on the following formula:

$$\text{LDH activity}=267.2\times ∆A,\left[\mu \text{kat}/{\text{dm}}^{3}\right]$$

where: Δ*A*–the absorbance change per minute.

Results from three trials were averaged. The value of the standard deviation was calculated.

#### 2.8.4 Morphology and cell culture visualization

After 0, 2, 4, 6, and 8 days of culture, nonwoven discs with cells were transferred to new 24-well plates and rinsed twice with 1 mL of DPBS, no calcium, no magnesium buffer. Next, the materials were soaked in: (1) 1 mL of 4% (w/v) aqueous paraformaldehyde solution (PFA, Sigma-Aldrich, DE), (2) 1 mL of 0.2% (v/v) aqueous Triton X-100 solution (Sigma-Aldrich, DE), (3) 200 μL of 165 nM Alexa Fluor 488 Phalloidin (Thermo Fisher Scientific, US) in DPBS solution, and (4) 100 μL of 300 nM DAPI (Thermo Fisher Scientific, US) in DPBS solution. After each step, the materials were rinsed twice with 1 mL of DPBS, no calcium, no magnesium buffer. Samples were visualized by an LSM 880 confocal laser scanning microscope (Carl Zeiss AG, Jena, DE).

## 3 Results and discussion

**SCHEME 1:**
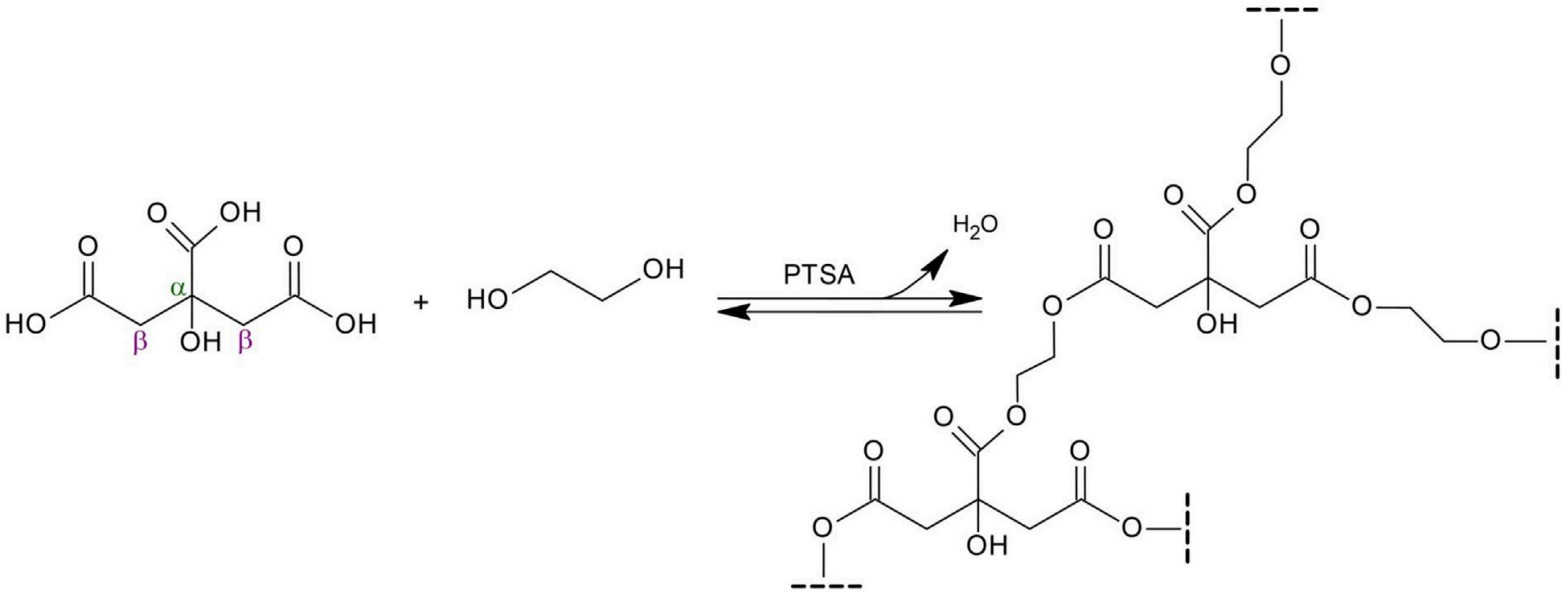
Synthesis of poly(1,2-ethanediol citrate) from citric acid and ethylene glycol.

Poly(1,2-ethanediol citrate) (PECit, Scheme 1) was synthesized and used to obtain nonwovens by electrospinning. Due to its poor fibre-forming properties, the addition of a carrier polymer, polylactide (PLA), was used in different mass percentages, resulting in the following types of nonwovens: 25%PECit, 50%PECit and 75%PECit.

### 3.1 Morphology of the nonwovens

The study aimed to investigate the effect of the PECit addition on the properties of PLA nonwovens. Poly-L-lactide is a hydrophobic material that adversely affects cell adhesion on biomaterials. At the same time, it exhibits good processability and can act as a carrier polymer.

**FIGURE 1:**
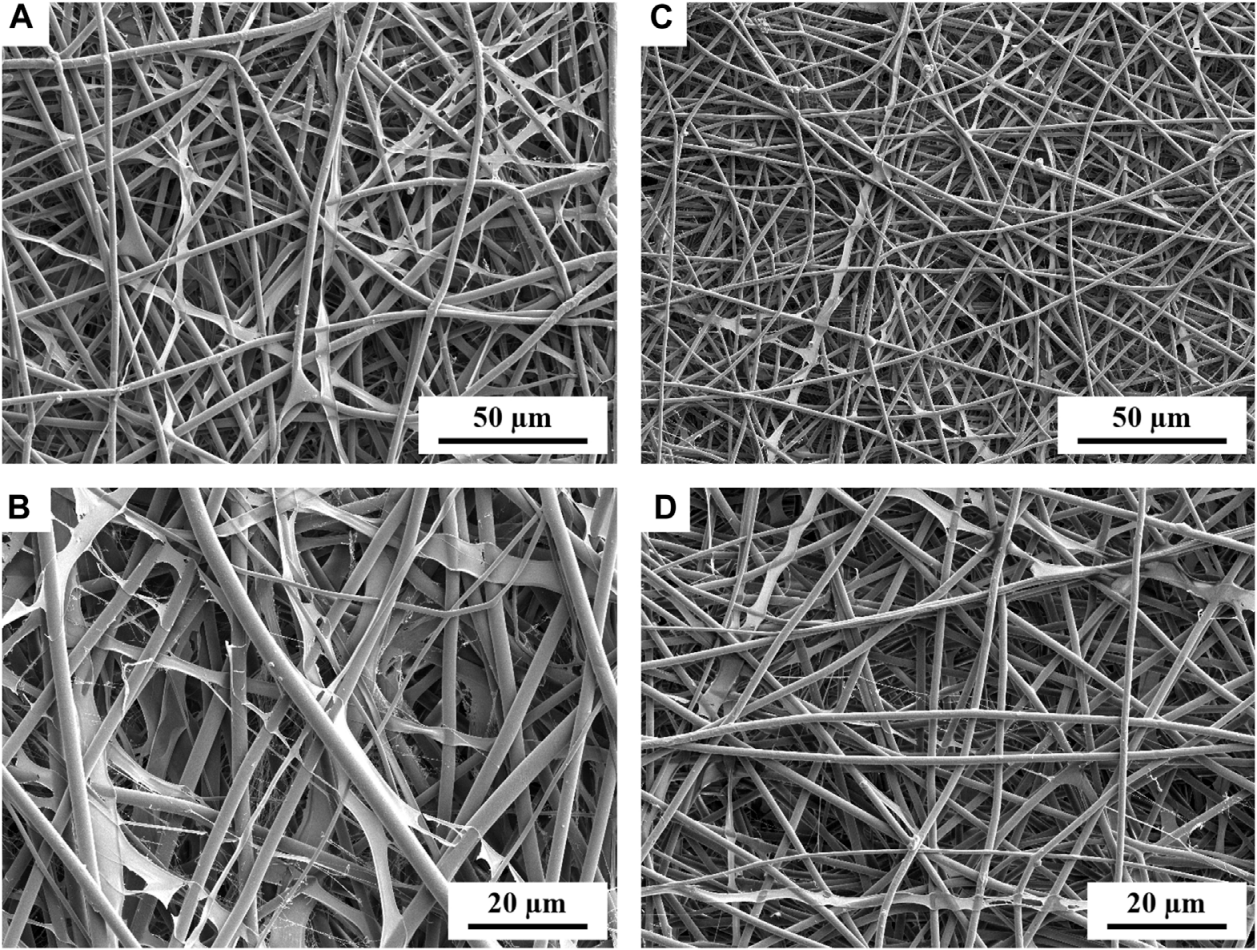
SEM micrographs showing the morphology of PLLA + PECit nonwovens: 25%PECit **(A,B)** and 50%PECit **(C,D)** at two different magnifications.

The 75%PECit nonwovens, despite the successful production of an electrospun sheet, showed inferior potential for further use due to their very high adhesion to any surface and the associated difficulties in their analysis and processing. Heterogeneities in their macroscopic structure were also noticeable. For this reason, their further examination was not pursued. The morphology of the other nonwovens was visualized with a scanning electron microscope (Figure 1).

In both cases, nonwoven sheets with fiber diameters ranging from 1 to 5 µm were obtained, with the average 50%PECit fibers being noticeably thinner (Figures 1C, D). Some structural defects are visible, including beads, local bends, torsions, and breaks. Compared to typical PLA fibers, which are rigid and straight, there is a high degree of disorder in PLA + PECit nonwoven structure and randomness in the arrangement of the fibers. At the presented rate, it should be considered a benefit since it mimics a pretty random structure of natural extracellular matrix in the tissue to some degree.

It can be seen that, despite the lower content of PECit, it forms locally flattened, not fully formed fibers (25%PECit, Figures 1A, B). Considering the heterogeneous form of the 75%PECit nonwoven sheet, too much excess of either polymer has a detrimental effect on the homogeneity of the mixture and consequently affects the efficiency of electrospinning. This is due to differences in the chemical nature of PLA and PECit (hydrophobic and hydrophilic). 50%PECit fibers are best formed. However, local defects were generally deemed to be of little consequence to the material properties, especially cell adhesion.

### 3.2 Surface hydrophilicity

The hydrophilic-hydrophobic properties of materials are relevant in determining their potential applications, especially for biomaterials. In general, an increase in surface hydrophilicity promotes cell adhesion and favors cell proliferation. Polylactide belongs to the group of hydrophobic materials with a water contact angle value of 125° (Montgomery et al., 2017). Adding poly(1,2-ethanediol citrate) should decrease the water contact angle of the material, i.e., increase hydrophilicity. Theoretically, an increase in PECit content is associated with introducing more carboxyl and hydroxyl functional groups, so an increase in the polycitrate content of the nonwoven fabric should proportionally increase the hydrophilicity of the material.

**FIGURE 2:**
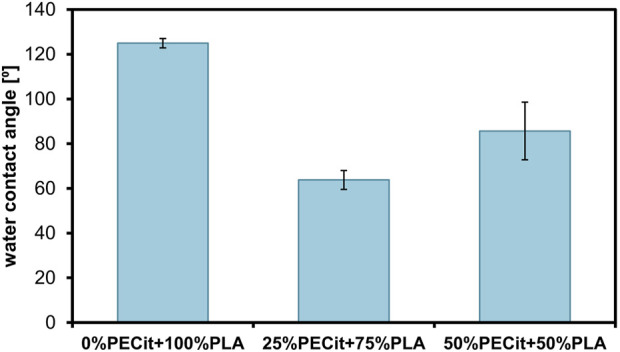
Water contact angles of the PLA and PLA + PECit nonwovens.

The results of water contact angle determination are presented in Figure 2.

It is apparent that the addition of PECit has a hydrophilizing effect on the PLA nonwovens; however, the nature of this impact might be unclear. To verify this statement additional experiments with gradient content of PECit may be conducted in the future. The higher mass percentage of polycitrate (50%PECit) does not correspond to a more hydrophilic surface compared to 25%PECit nonwoven, as the values are (85,7 ± 12,9)° and (63,8 ± 4,2)°, respectively. However, the difference between these materials and PLA nonwovens (125⁰) is apparent.

Given the previous reflections on the homogeneity of the polymer blend, it can be concluded that the mass content of polycitrate does not directly translate into the surface area content. Still, the desired result was achieved overall, as the studied nonwovens exhibit better hydrophilicity than those made from PLA.

### 3.3 Mechanical and thermal properties

Characterization of the mechanical properties of PLA + PECit nonwovens was carried out by static tensile testing. Based on the results obtained for each material, toughness, tensile strain at break, and tensile modulus were determined. The calculated values are summarized in Table 1.

**TABLE 1:** Tensile test results.

	25%PECit	50%PECit
Tensile modulus [MPa]	84.6 ± 15.5	48.5 ± 10.8
Tensile strain at break [%]	228.0 ± 21.0	89.4 ± 14.3
Toughness [mJ/mm^3^]	6.41 ± 0.94	1.61 ± 0.39

There is a noticeable decrease in the tensile strength of the materials as the PECit content increases. This is related to the chemical structure of this polyester, which, as a highly branched molecule, does not reach high molecular weights during the synthesis of the prepolymer resin, which is then used for electrospinning. After reaching a certain critical value of substrate conversion, the reaction mixture could gel and result in a product of infinitely high molecular weight. However, such a gelled polymer would not be suitable for further processing due to its insolubility in organic solvents.

The tensile strength of a polymer increases with its molecular weight. Long polymer chains become entangled resulting in better resistance to deformation before tearing. Therefore, a decrease in mechanical strength is inevitable when PLA is doped with oligomers. Still, it is a material with potential applicability for culturing soft tissue cells, such as skin (Kalra and Lowe, 2016; Kalra and Lowe, 2016). Depending on the mass content of the polycitrate, Young’s tensile modulus value of the material can be adjusted to match the parameters of any skin tissue.

**FIGURE 3:**
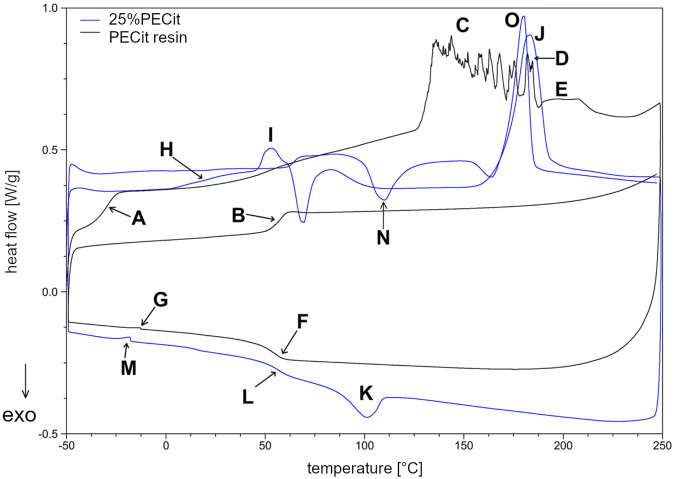
DSC thermogram of PECit prepolymer resin and 25%PECit nonwoven.

**FIGURE 4:**
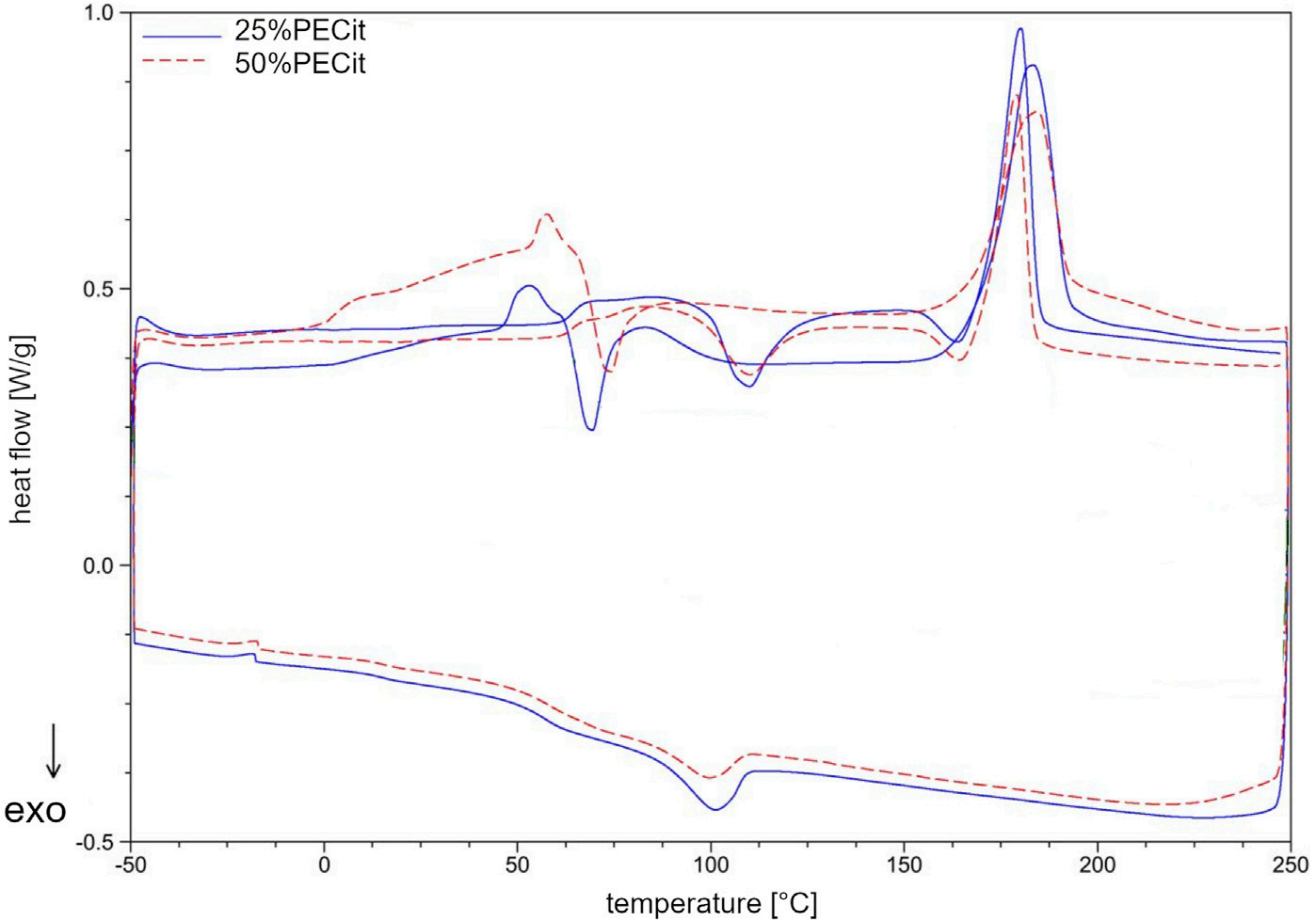
DSC thermogram of 25%PECit and 50%PECit nonwovens.

Thermal analysis was carried out using differential scanning calorimetry (DSC). The glass transition (*T*_g_), cold crystallization (*T*_cc_) and melting (*T*_m_) temperatures and the degree of crystallinity (*X*_c_) for PLLA were determined. Thermal analysis was carried out in two heating cycles to study the properties of the materials before and after removing their thermal history. Results are presented in Table 2; Figures 3, 4.

**TABLE 2:** Degree of crystallinity of the nonwovens.

	First heating			second heating		
	T_m_ [°C]	ΔH_m_ [J/g]	X_c_ [%]	T_m_ [°C]	ΔH_m_ [J/g]	X_c_ [%]
25%PECit	182.99	55.19	59.3	180.40	30.24	32.5
50%PECit	184.51	48.30	51.9	179.31	16.85	18.1

The DSC curve (Figure 3) shows a glass transition temperature at −29.52°C (A) during the first heating of the resin sample. In the second heating, the temperature of the phase transition of the glassy state to the elastic state is shifted to 57.82°C (B). The significant increase in the glass transition temperature may be due to the conversion of free functional groups and a decrease in the mobility of polymer chains, resulting in more energy required for the transition to the elastic state. Another rationale is the effect of the physical ageing of the polymer under the influence of temperature. During the first heating, in the temperature range from about 50°C to 125°C, a continuous increase in heat uptake can also be observed, which indicates the relaxation of the polymer chains and the mild reactivation of the free functional groups. Above 125°C (C), endothermic effects indicate rapid reactions between free functional groups and reaching the critical point of the polycondensation (gelling). From about 170°C (D) on, the decarboxylation effect of unreacted citric acid and the decomposition of the polymer structure are also superimposed in the temperature range in question. In the temperature range from 188.61°C to 228.62°C (E), a broad endothermic effect corresponding to the evaporation of unreacted ethylene glycol can also be observed. In the second heating, the discussed impacts do not occur. During cooling at 55.56°C (F), a return to the glassy state can be observed. At −12.75°C (G), there is a slight endothermic effect, which with high probability may indicate the presence of a second phase of PECit, with lower molecular weights, which was not crosslinked during the first heating.

In the nonwoven fabric, the temperatures characteristic of the physical transformation of the polycitrate are altered. In the first heating, the glass transition temperature of PECit cannot be precisely determined but probably shifts to about 10°C (H). From 20°C to 46°C, stress relaxation of the polymer chains in the nonwoven can be observed. At 46.21°C (I), the glass transition temperature of PLLA is visible, after which up to about 83°C an exothermic effect associated with a change in the heat capacity of the test material is observed. Melting of PLLA’s crystalline phase can be observed at 182.99°C (J). During cooling at 100.23°C (K), PLLA crystallization occurs, followed by glass transition at 57.21°C (L). At −17.63°C (M), there is a slight endothermic effect due to two PECit fractions. Mixing with PLLA increases the signal relative to pure polycitrate (M) due to separating the PECit fractions and less homogenizing the polymer structure. In the second heating, a small endothermic effect is seen at 62.00°C, most likely corresponding to the glass transition temperature of PLLA.

The cold crystallization of PLLA can be seen in the thermogram as an exothermic peak at 109.88°C (N). The melting point of PLLA decreased from the first heating to 180.40°C (O). The melting of PLLA precedes the exothermic effect associated with the reorganization of the structure of the polymer chains.

Based on the DSC curves of PLLA + PECit nonwovens (Figure 4), one can conclude that the thermal properties of materials containing 25% and 50% PECit by weight are similar. The change in the proportion of polymers affects the reduction of peaks corresponding to the phase transformations of the polylactide. In addition, the heat capacity of the material decreases significantly. A broad exothermic effect appears in the temperature range from 75°C to 150°C, which probably corresponds to the temperature region of PECit flow. Thermal analysis of PLLA + PECit nonwovens indicates that at 37°C, PECit is entirely amorphous.

Based on the thermograms obtained, the degree of crystallinity of PLLA in nonwovens was also calculated (Table 2). It was found that an increase in the content of PECit in the polymer mixture affects the decrease in the degree of crystallinity of PLLA. This is due to hindered crystallization of the polylactide chains and reduced mobility by closer proximity to the amorphous polycitrate. However, changing the mass ratio of polyesters does not significantly affect the phase transition temperatures. For both materials, the polylactide exhibits crystalline features (the presence of visible *T*_g_, *X*_c_>50%). When the thermal history is removed, the degree of PLLA crystallinity is reduced.

### 3.4 Cytotoxicity

**FIGURE 5:**
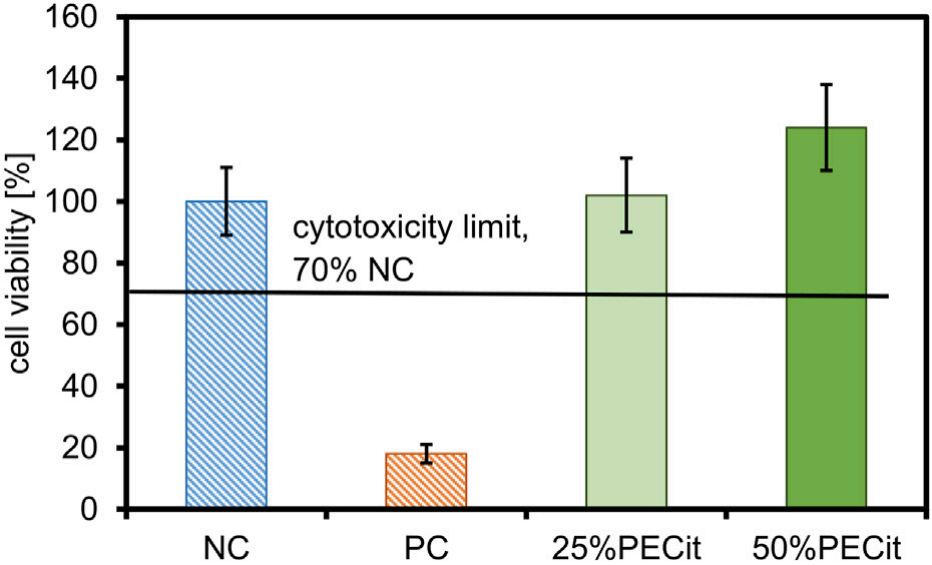
The XTT cytotoxicity test results.

The cytotoxicity effect of the PLA + PECit nonwovens was assessed by the results of the colorimetric XTT reagent assay. Cell viability evaluation was based on the spectrophotometrically measured absorbance at 450 and 660 nm, following ISO EN 10993-5: 2009. The results of the XTT cytotoxicity test are presented in Figure 5.

The first step in determining the biocompatibility of a material is the assessment of cytotoxicity, which, according to the accepted limit, should be no less than 70% cell survival of the negative control (Bandzerewicz et al., 2023). The colorimetric XTT assay is commonly used to assess the cytotoxicity, in which active mitochondria reduce the XTT reagent to orange-colored formazan.

Based on the positive control result, it can be concluded that the procedure for assessing cytotoxicity was performed correctly. All PLLA + PECit nonwovens are non-cytotoxic. For nonwoven 25%PECit, the difference in cell viability compared to the negative control is not statistically significant. For the 50%PECit nonwoven, cell survival is higher than 100%, which also indicates the absence of cell dysfunction. However, it should be emphasized that the result in this case should be interpreted cautiously and does not indicate the effect of promoting cell growth by PECit. The result obtained may be the result of: (1) non-specific reduction of the XTT reagent by com-pounds interacting with the dye, thus inflating the absorbance reading, (2) technical errors, including, but not limited to, an error during the procedure, inhomogeneous dilution of the cell suspension or inaccurate reading from the microplate reader.

Based on the results obtained, it can be concluded that the PLLA + PECit nonwovens are non-cytotoxic. Additional tests were conducted to determine the influence of modifying PECit nonwovens on line L929 cells.

### 3.5 L929 cell cultures

**FIGURE 6:**
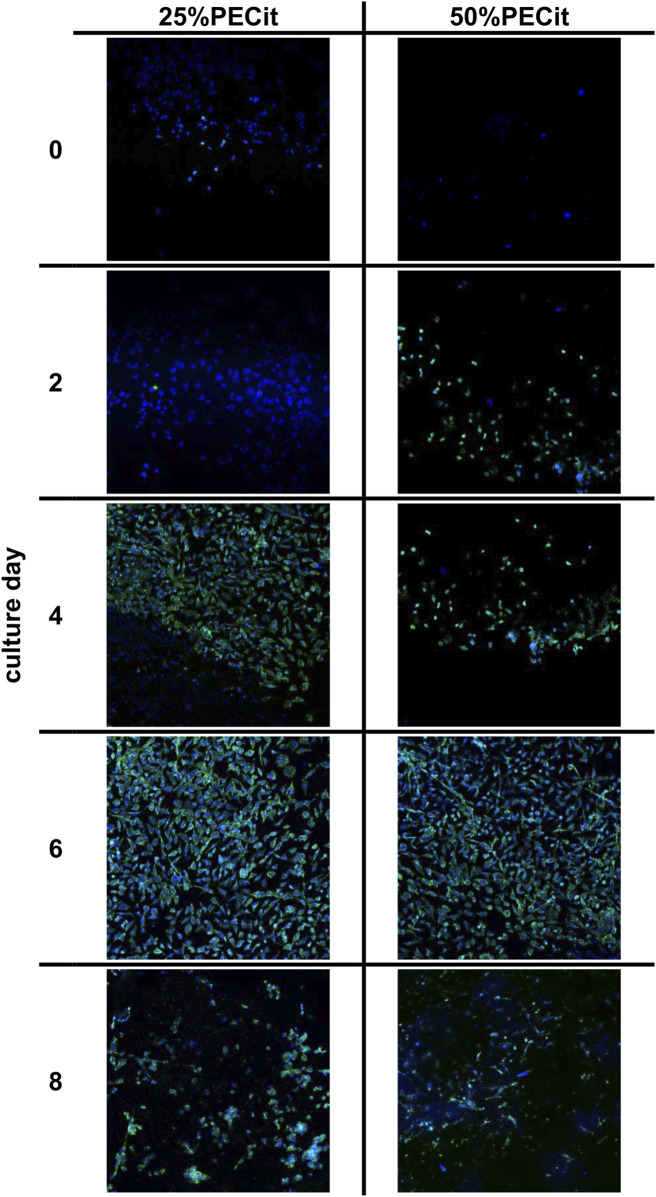
Morphology assessment of L929 cells on nonwovens using confocal microscopy.

**FIGURE 7:**
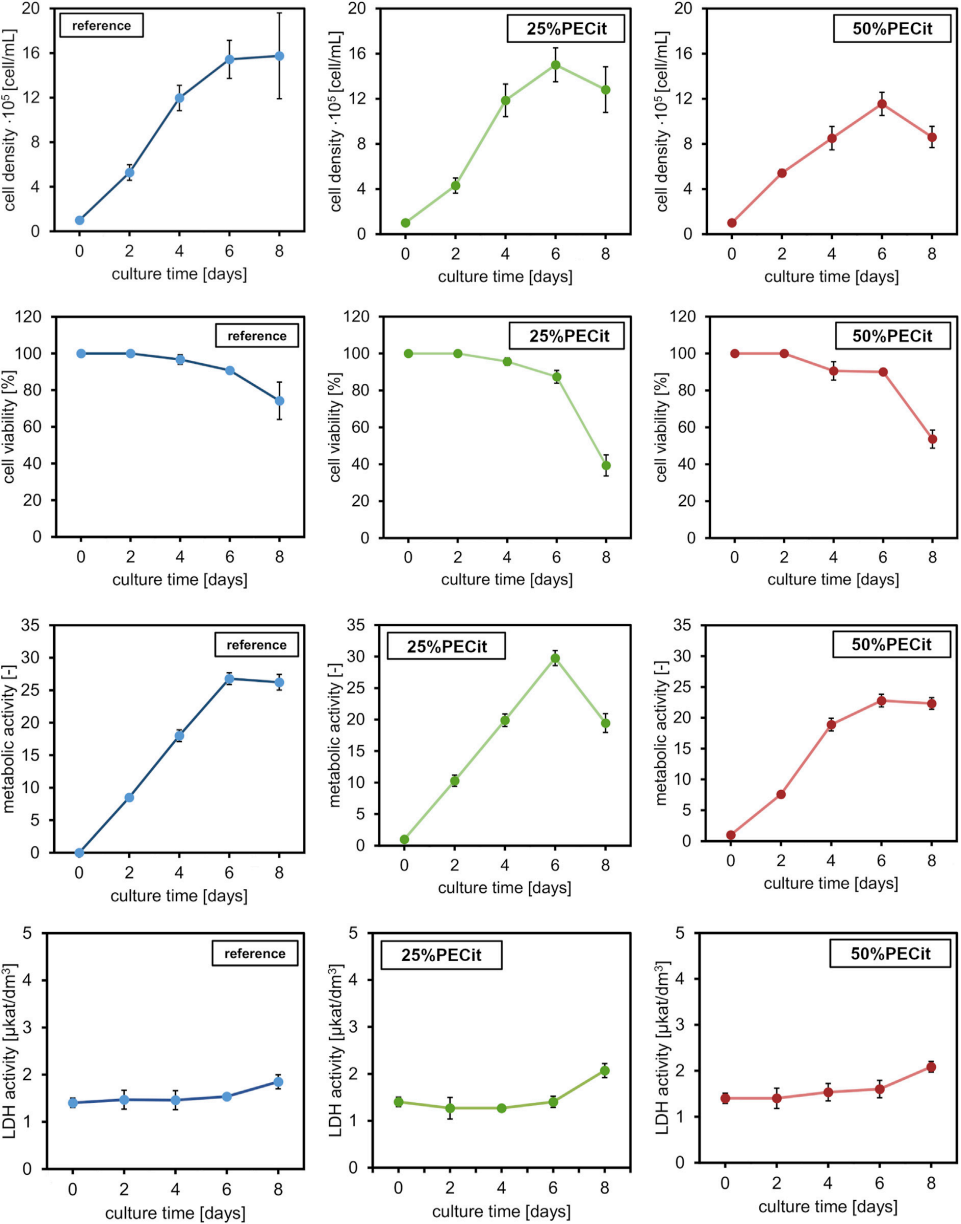
L929 culture properties at different time points: cell density, cell viability, metabolic ac-tivity, and LDH activity. Comparison between the reference culture and cells cultured on the nonwovens.

L929 cells were maintained for 8 days without medium exchange, and samples were harvested every 48 h. Images of cells from the confocal microscope are presented in Figure 6. The actin skeleton is stained green, and the nuclei are stained blue. Figure 7 shows changes in different culture parameters for all the nonwovens compared to the reference culture.

At day 0, a difference in cell density is observed on materials with different PECit content in the nonwoven fabric. However, it can be assumed that this effect is related to the local formation of cell colonies and is not directly due to the different content of polycitrate.

After 2 days of culture, the density of cells on both materials is similar. The spherical morphology and lack of visible nuclei indicate an early stage of mitotic cell divisions. After 4 days of culture, the cells are already shaped into typical spindle-shaped structures with clearly developed building structures. Cell adhesion to a nonwoven fabric containing an equal mass ratio of polymers appears slower on confocal microscopy images. Cells likely proliferate at different rates due to differences in pH level of culture medium. For the culture containing PLLA + PECit nonwoven with 50% PECit the acidification of the environment may be faster due to the higher content of polycitrate in the medium, resulting from the degradation of nonwovens. However, the observed cell density in both materials on day 6 of culture is similar. Confocal microscopy images of L929 cultures for this day show that cells covered almost the whole surface of the tested materials, which means that the confluence of cultures is achieved. These results confirm the lack of cytotoxicity of the studied materials and indicate that the surface of both materials provides favorable conditions for L929 cell growth. Observed on day 8, the decrement in cell density is probably caused by the limitation of space for cell proliferation and the consequent apoptosis that follows.

Typical curves showing cell density were obtained for all materials in which no adaptation (lagging) phase is observed. The lack of a lagging phase probably resulted from low water contact angle values (hydrophilicity of the materials), which facilitated cell adhesion to the materials. The exponential growth phase lasts until day 6. The growth curve of cells of the L929 line in the reference sample corresponds to the literature results (Gadomska-Gajadhur et al., 2021; Ulatowski et al., 2022). For the reference culture, there is a stationary phase between 6 and 8 days. During this time, a decline phase of fibroblasts can be observed, i.e., aging and cell death. The discussed relationship is related to reducing the space necessary for further cell proliferation. Analogous conclusions were also presented when interpreting confocal microscope images and can be confirmed by the decrease in viability of cultures after day 6 of culture. Furthermore, the lower value of cell density of L929 cells on 50%PECit nonwoven than on 25%PECit nonwoven, both noticed on day 4, is consistent with the results of confocal microscope analysis (Figure 6, day 4).

By day 6, the viability of cells on the 25%PECit nonwoven fabric is comparable to that of the reference sample. A decrease in cell viability of about 10 percentage points can be observed in the case of material with a higher polycitrate content between days 2 and 4. This is probably due to the slight acidification of the culture medium by the products of nonwoven degradation.

The metabolic activity of all cultures reaches a maximum value on day 6 of culture. By day 6, the highest metabolic activity is shown by cultures on the 25%PECit filament. The higher polycitrate content reduces the cells’ metabolic activity to a level comparable to or lower than that of the reference sample. By day 8 of culture, a slight decrease in metabolic activity is evident in the two materials. At the same time, the sharp decline in 25%PECit again indicates inhibition of proliferation due to excessive cell density. Acidification of the culture medium to a cell-killing level is unlikely in this case due to the absence of the discussed effect at higher polycitrate contents.

The activity of lactate dehydrogenase (LDH) leaking from damaged cells for cultures grown on 25%PECit nonwovens up to day 6 is lower than the reference sample’s activity level. However, the LDH activity increase between days 6 and 8 for both materials, consistent with the results presented for cell density and viability. For 50%PECit, LDH activity exceeds the reference sample as early as day 4. It should be noted that the LDH activity for both materials is insignificant, indicating a lack of negative impact of the tested materials on L929 cells.

Studies have shown that both nonwovens provide favorable conditions for the growth of L929 cells. However, slight differences in cell proliferation were observed between two tested materials. These could be due to differences in the nonwovens’ wetting angle, morphology or mechanical properties. Higher values of water con-tact angle, lower values of mechanical parameters and thinner fibers of the 50%PECit nonwoven than for the 25%PECit nonwoven, may have been the reason for the lower values of parameters characterizing biomass proliferation. In addition, the higher PECit content in the 50%PECit nonwoven, may have caused faster acidification of the medium, which may have negatively affected the cultured L929 cells. However, further studies are required to analyze in detail the effect of PECit content in the material on L929 cells.

Comparing the results with the study performed by Ulatowski et al., 2022, it can be observed that the reference culture on the 6th day of culture in Ulatowski et al., 2022 article had a lower concentration of cells on the surface than the culture on these materials. The maximum concentration of cells, i.e., the confluence of culture, was observed in this study on the 6th day of culture and the 8th day of culture in reference culture performed by Ulatowski et al., 2022. These results suggest that the materials used in this study provide better conditions for L929 cells than the surface growth typically used to maintain adherent cells. In our opinion, the faster growth of cells probably caused the space limitation for cell proliferation and the consequent apoptosis that follows. It seems to us that cell apoptosis did not occur as a result of the toxicity of the tested materials, as this effect would have already been evident in the earlier days of culture, which were characterized by a higher number of cells than the reference culture presented in the article by Ulatowski et al., 2022.

## 4 Conclusion

In the present work, we successfully synthesized a polymer based on citric acid and 1,2-ethanediol, then used it to obtain porous nonwovens by electrospinning from a mixture with polylactide. Based on the SEM images, it was found that the material containing an equal mass ratio of polymers had the best morphology. Changing the PECit content can result in the formation of local structural defects, including beads. Too high a PECit content results in materials that are difficult to use and apply, indicating that this polycitrate can serve mainly as a maximum 50% additive to another carrier polymer, acting as a modifier of the properties of such polymer. 25% and 50%PECit materials show a degree of porosity suitable for cell colonization.

The hydrophilic-hydrophobic characteristics of the nonwovens indicate that hydrophilic materials were obtained. The hypothesis of a proportional increase in the wettability of the material with an increase in the mass proportion of PECit in the material was not confirmed. An increase in the PECit content of the nonwoven results in a lower heat capacity of the material and a decrease in the degree of PLLA crystallinity. Thermal analysis of nonwovens indicates that at 37°C PECit is entirely amorphous. Depending on the mass content of the polycitrate, Young’s modulus value of the material can be adjusted to match the mechanical parameters of any type of skin tissue.

Based on the most important parameters for evaluating the condition of cultured L929 cells (cell density and viability, metabolic activity and LDH), the potential of PLLA + PECit nonwovens for tissue engineering applications was established.

## Data Availability

The datasets presented in this study can be found in online repositories. The names of the repository/repositories and accession number(s) can be found in the article/Supplementary Material.
